# Fatty Acids, CD36, Thrombospondin-1, and CD47 in Glioblastoma: Together and/or Separately?

**DOI:** 10.3390/ijms23020604

**Published:** 2022-01-06

**Authors:** Cristiana Tanase, Ana Maria Enciu, Elena Codrici, Ionela Daniela Popescu, Maria Dudau, Ana Maria Dobri, Sevinci Pop, Simona Mihai, Ancuța-Augustina Gheorghișan-Gălățeanu, Mihail Eugen Hinescu

**Affiliations:** 1 Victor Babes National Institute of Pathology, 050096 Bucharest, Romania; ana.enciu@umfcd.ro (A.M.E.); elena.codrici@ivb.ro (E.C.); daniela.popescu@ivb.ro (I.D.P.); maria.dudau@ivb.ro (M.D.); ana-maria.dobri@drd.umfcd.ro (A.M.D.); spop@ivb.ro (S.P.); simona.mihai@ivb.ro (S.M.); mhinescu@yahoo.com (M.E.H.); 2 Department of Cell Biology and Clinical Biochemistry, Faculty of Medicine, Titu Maiorescu University, 031593 Bucharest, Romania; 3 Department of Cell Biology and Histology, Carol Davila University of Medicine and Pharmacy, 050474 Bucharest, Romania; agheorghisan.a@gmail.com; 4 Department of Neurology, National Institute of Neurology and Neurovascular Diseases, 077160 Bucharest, Romania; 5 ‘C.I. Parhon’ National Institute of Endocrinology, 001863 Bucharest, Romania

**Keywords:** fatty acids (FA), CD36, CD47, thrombospondin-1 (TSP-1), glioblastoma (GBM)

## Abstract

Glioblastoma (GBM) is one of the most aggressive tumors of the central nervous system, characterized by a wide range of inter- and intratumor heterogeneity. Accumulation of fatty acids (FA) metabolites was associated with a low survival rate in high-grade glioma patients. The diversity of brain lipids, especially polyunsaturated fatty acids (PUFAs), is greater than in all other organs and several classes of proteins, such as FA transport proteins (FATPs), and FA translocases are considered principal candidates for PUFAs transport through BBB and delivery of PUFAs to brain cells. Among these, the CD36 FA translocase promotes long-chain FA uptake as well as oxidated lipoproteins. Moreover, CD36 binds and recognizes thrombospondin-1 (TSP-1), an extracellular matrix protein that was shown to play a multifaceted role in cancer as part of the tumor microenvironment. Effects on tumor cells are mediated by TSP-1 through the interaction with CD36 as well as CD47, a member of the immunoglobulin superfamily. TSP-1/CD47 interactions have an important role in the modulation of glioma cell invasion and angiogenesis in GBM. Separately, FA, the two membrane receptors CD36, CD47, and their joint ligand TSP-1 all play a part in GBM pathogenesis. The last research has put in light their interconnection/interrelationship in order to exert a cumulative effect in the modulation of the GBM molecular network.

## 1. Introduction

Glioblastoma (GBM) is one of the most aggressive tumors of the central nervous system, characterized by a wide range of inter- and intratumor genetic heterogeneity [1,2,3] and extensive epigenetic mechanism dysregulations [4,5]. Like most cancers, GBM rewires its metabolism towards lipolysis, in order to provide energy and to generate ATP and macromolecules necessary for tumor cell growth, division, and survival [6]. Accumulation of fatty acids (FA) metabolites and down-regulation of oxidation enzymes were associated with low survival rates in high-grade glioma patients [7,8,9,10,11]. In the past decade, the ‘metabolic switch’ in cancer cells had been intensely studied, starting with the relationship between cancer genes and metabolic alterations and continuing with unveiling some other metabolic processes which may have an important contribution to cancer cells function such as FA synthesis and oxidation [12]. In addition, the FA may contribute as donors to acetyl and methyl group pools required for epigenetic modification, and accordingly could be capable to modulate epigenetic mechanisms [12,13,14]. The brain is the second richest organ in lipids besides adipose tissue, with about 50% of its dry weight represented by lipid content [15,16]. The diversity of brain lipid categories is greater than in all other organs [17], with polyunsaturated fatty acids (PUFAs) representing about 35% of all brain lipid components [18]. Several classes of proteins, such as FA transport proteins (FATPs) and FA translocases, are considered principal candidates for PUFAs transport through BBB and delivery of PUFAs to brain cells. Of these, the CD36 FA translocase promotes long-chain FA uptake as well as oxidated lipoproteins (including those forming low-density lipoproteins-LDL) [19]. CD36 also binds and recognizes extracellular matrix proteins such as thrombospondin-1 (TSP-1) and proteins that contain the TSP-1 structural homology region (TSR), fibronectin, and collagen [20]. TSP-1 was shown to play a multifaceted role in cancer as part of the tumor microenvironment. Besides its effects on tumor cells, TSP-1 also affects tumor stromal cells such as endothelial cells, fibroblasts, macrophages, dendritic cells, and T cells. These effects are mediated by TSP-1 not only through the interaction with CD36 but also CD47, a member of the immunoglobulin superfamily [21,22].

Separately, FA, the two membrane receptors CD36, CD47, and their joint ligand TSP-1 all play a part in GBM pathogenesis, as discussed in the following sections. A question that has not been yet addressed is whether they intersect or exert a summative effect in the GBM molecular network, which will be the main focus of this review.

## 2. Fatty Acids, CD36, Thrombospondin-1, and CD47 in Glioblastoma Development and Progression

### 2.1. Fatty Acids

Cancer-associated metabolic changes include, among other hallmarks [23], the altered lipid metabolism, which includes dysregulations in FA transport, de novo lipogenesis, storage of FA as lipid droplets (LDs), and β-oxidation to generate ATP [24,25].

GBM is one of the most rapidly growing malignant tumors with an enormous demand for energy and biomass to fuel tumor development and progression. Furthermore, in GBM, the bio-energetic pathways are interconnected to pro-oncogenic signaling. AMPK signaling is linked with catabolism and cell cycle progression, mTOR signaling supports cancer metabolic flexibility and cancer cell survival, and the electron transport chain yields ATP and reactive oxygen species (ROS), which act as signaling molecules [26,27]. Mutations in the tumor suppressor p53 and the tricarboxylic acid cycle enzymes IDH 1 and 2 are involved in diverse oncogenic signaling and contribute to establishing metabolic phenotypes in GBM [28,29]. In GBM, p53 is frequently mutated, as the progression from low to high malignant phenotypes of gliomas is characterized by the selection of permissive mutations or deletions [30]. Therefore, interactions between p53 and target genes most likely differ with the protein phenotype. Moreover, GBM as a heterogeneous malignancy has a diverse tumor microenvironment that includes regions of hypoxia, necrosis, and differential nutrient gradients within an individual tumor [6,31]. Accordingly, the GBM cells may adapt their metabolic choices based on the type of surrounding environment.

GBM cells have unusually high levels of free fatty acids; therefore, lipid homeostasis regulates lipid droplets formation to prevent oxidative damage. DGAT1 is an enzyme involved in lipid storage organelles formation and TGs being upregulated in GBM, which prevents lipotoxicity [32]. Currently, emerging evidence suggests that GBM can synthesize de novo significant amounts of non-essential FA [7,31,33]. Mainly, the synthesis of FA depends on cytosolic acetyl-CoA generation by using substrate glucose or acetate and going through the TCA cycle. The reactions can be limited by acetyl-CoA synthetase (ACSS) expression. Indeed, in astrocytes by concurrent insertion of a BRAFV600E mutation and deletion of TP53 and PTEN, which are common oncogenic alterations in GBM, the expression of ACSS2 increased seven-fold and accordingly the cytosolic pool of acetyl-CoA [34]. In addition, the knockdown of ACSS2 decreased GBM neurosphere growth and viability; this fact can be explained by the decreased oxidation rate of acetyl-CoA in the mitochondria [34].

It has been demonstrated that gliomas are enriched in ω-6 PUFAs, arachidonic acid (AA), and its precursor, linoleic acid (LA), but not ω-3 PUFASs [35]. Whilst the brain can synthetize de novo most of the saturated and monounsaturated fatty acids, it predominantly lacks the capacity to synthetize some PUFAs [36,37], such as LA, relying on food intake as the main source. Increased AA concentration in the extracellular environment was associated with increased mobility of malignant glioma cells [9], while downstream metabolization of AA on eicosanoid and leukotrienes pathways was proposed as fueling mechanisms for glioma tumors [38]. Conversely, unmetabolized AA leads to the accumulation of ceramide, a second messenger and potent activator of intrinsic apoptosis [39,40].

GBM cells U138-MG were also enriched in palmitic acid (C16:0), stearic acid (C18:0), and oleic acid (C18:1) [41]. LC-MS metabolomic analysis using fully labeled 13C palmitic acid showed that this saturated FA fuels the tricarboxylic acid cycle to produce ATP [42].

As for cholesterol, an important structural component of membranes, it is known that brain cholesterol metabolism is very different from the rest of the body (reviewed in [43]), with astrocytes as the main site of cholesterol synthesis. Furthermore, cholesterol esterification and lipid droplet formation were proposed as markers of GBM [44]. Interference with cholesterol metabolism was proposed as a therapeutic option for GBM treatment (reviewed in [45]) based on data accumulated over time, as early as the 1990s, showing the accumulation of cholesterol esters in glioma tissue and surrounding regions [46]. Recently, statins drugs commonly used to inhibit cholesterol biosynthesis have been even proposed as repurposing drugs to fight cancer [47,48].

EGFR inhibitor lapatinib treatment on a GBM patient induces the cleavage and nuclear translocation of a master transcriptional regulator of fatty acid synthesis, sterol regulatory element-binding protein 1 (SREBP-1) [49]. Moreover, in vivo studies have shown that GBMs, without constitutively active EGFR signaling, were resistant to the inhibition of FA synthesis, whereas the insertion of vIII mutation sensitized tumor cells in GBM xenograft models [50]. Importantly, by targeting key enzymes of FA synthesis pathways, such as SREBP-1, acetyl-CoA carboxylase ACC, and FA synthase (FASN), or by blocking the fatty acid elongase 2 (ELOVL2), which catalyzes the elongation of fatty acids, glioblastoma cell growth, and the inhibition of tumor initiation or promotion of apoptosis [50,51,52].

Hypoxic regions are a common feature of GBM that defines its aggressiveness and has been associated with resistance to chemotherapy and radiation as well as tumor invasion and poor patient survival [53,54]. Additionally, hypoxia is considered to have a major contribution to the metabolic reprogramming of cancer cells [55]. In hypoxia, the possible reduced FA synthesis observed is compensated by increased uptake of exogenous lipids, mainly monounsaturated acyl lipids [25].

The hypoxic environment of a tumor leads to the stabilization of the hypoxia-inducible factor-1α (HIF-1α), which stimulates glycolysis and also inhibits mitochondrial respiration [31,56]. A study on in vivo model of antiangiogenic therapy has shown increased lipid droplets (LDs) accumulation in a HIF-1α-dependent manner by increasing FA binding proteins 3 and 7 (FABP3 and FABP7) expression, rather than *de novo* FA synthesis. Upon re-oxygenation, tumor cells used the LDs for energy production and antioxidant defense [56]. In normal cells, lipid droplets are energy storage organelles with much accumulation in hepatic and adipose tissues, but recent studies reported large amounts of LDs in tumor tissues, including GBM [44]. Likewise, it has been reported that under lipid-limited growth conditions, tumor cells can switch to the de novo FA synthesis in order to maintain their growth and survival [33].

Recently, the global metabolomic analysis coupled with gene expression profiling on patient-derived gliomas identified fatty acid β-oxidation (FAO) as a dominant metabolic node in GBM [24]. The study highlighted that the enhanced FAO is directly dependent upon the tumor microenvironment, establishing the metabolic fate of GBM cells. In nutrient-favorable conditions, the FAO drives the GBM proliferation, while in a nutrient-unfavorable environment, it acts as an alternate source of ATP [24].

Another study suggests that in GBM, FAO can be a major provider of the cell’s acetyl-CoA pool, instead of glucose glycolysis [57]. FAO is also a source of important lipid secondary metabolites and provides acetyl groups for protein and histone modification and NADPH [12]. Recently, Duman et al. demonstrated that the acyl-CoA-binding protein (ACBP) drives GBM growth by promoting mitochondrial long fatty acyl-CoA accumulation and FAs β-oxidation [58]. Mechanistic experiments confirmed that the suppression of GBM cell proliferation rate can be induced by ACBP depletion. Thereby, this study established that ACBP may represent one of the crucial links between lipid metabolism and GBM progression [59]. In vitro assays have revealed FA utilization throughout the GBM metabolome and growth inhibition in GBM cell lines or patient-derived glioma cells treated with FAO inhibitors [42]. In addition, in vivo experiments have shown that knockdown of carnitine palmitoyltransferase 1A (CPT1A), which is the rate-limiting enzyme for FAO, or diacylglycerol-acyltransferase 1 (DGAT1), responsible for storing excess FAs in lipid droplets [32], reduce the rate of tumor growth and increased survival in xerograph mouse GBM model [32,42].

The interconnection between metabolism, tumor heterogeneity, and plasticity in GBMs has been demonstrated in stress conditions induced by radiation or chemotherapies. The treatment with standard therapy of temozolomide (TMZ) and radiation leads to alterations of GBM cells and drives them towards the GSC state [60,61]. The GSC population exhibits high levels of lipids and employs FA to resist in poor nutrient environments [62]. Using patient-derived xenograft cells, mouse models, transcriptomics combined with metabolic analyses, Caragher et al. found that GBM cells fate changes are followed by intensive changes in cells metabolic phenotype [63]. Furthermore, treatment with TMZ forced GBM cells to increase FA uptake both in vitro and in vivo in the plasticity-driven GSC population [63]. In conclusion, emerging evidence unveils the multiple facets of glioblastoma, the wide-range heterogeneity at the genetic and epigenetic levels, and the newly added metabolic complexity.

The relationship between lipid metabolism and GBM development is complex and repeatedly interrogated (reviewed in [7,26]), as well as the involvement of cholesterol [64] or that of certain lipids such as gangliosides [65]. This is not the focus of the present review—which addresses it only marginally—due to the involvement of CD36, a transmembrane scavenger receptor, in long-chain fatty acid translocation. Located at the interface between the cell and its environment, CD36 performs multiple tasks, of which those relevant to GBM will be discussed in the following sections.

### 2.2. CD36

CD36, a member of the class B scavenger receptor family is an 88-kDa transmembrane glycoprotein receptor responsible for many cellular events, from internalization or phagocytosis of the ligand to pro-inflammatory response [66,67]. The function versatility of CD36 is related to its ability to bind various ligands, from long-chain Fas mostly in liver and muscle to oxidized lipoproteins in the macrophage cell membrane and extracellular matrix (ECM) molecules such as TSP-1 or collagens (type I and IV). Almost all functions of CD36 have been found to be relevant to GBM tumor progression. CD36 protein is expressed at high levels in GBM in the presence of microglia/macrophage or endothelial phenotypic cells markers, but the same group identified a GBM cell population both negative for these markers but positive for CD36 in patient-derived tumors and patient-derived GMB xenografts [68]. The perivascular compartment, a CSC niche, is enriched in CD36, and the level of this protein transporter decreases with differentiation [68]. CD36 is reported to have an increased expression in glioma cancer stem cells (CSC) [68], along with integrin alpha 6 [69] and CD133 [70], previously known as CSC markers. CD36 ligands, particularly oxLDL, increase CSC proliferation, lipogenesis being critical for GBM cell growth [41]. Noting the fact that cancer stem cells have a high expression of CD36 and increased polyunsaturated fatty acid levels [71], this scavenger receptor could be related to a self-renewal and survival mechanism of glioma cells. Higher levels of this protein are linked with poor prognosis in both glioma and GBM tumors [68].

Activation CD36 in GBM has been related to inflammatory processes triggered by cellular debris released from the tumor site [72,73], notably induced by microglia and macrophages, key components of the tumor microenvironment [74]. Tumor-associated macrophages (TAMs) are abundant in gliomas [75,76]. Blood-derived TAMs, compared with microglial TAMs, show an altered oxidative metabolism with increased immunosuppressive cytokines expression, consequently promoting tumor growth, metastasis, ainducinguce drug resistance [77]. CD36 is a key receptor involved in lipid uptake and scavenger receptor for oxidized lipoproteins [78], involved in TAM generation in low-grade glioma and glioblastoma multiforme. Recent studies suggested that differentiation and activation of macrophages as pro-tumoral M2-TAMs is secondary to STAT6 phosphorylation, STAT6 being a target of FAO. Blocking or knocking down CD36 or inhibiting FAO in macrophages can impede TAM formation both in vitro and in vivo [79,80].

CD36 is also related to endothelial apoptosis via binding to TSP-1, exerting a strong antiangiogenic effect, with significance on tumor progression [81]. Vasculostatin, a 120-kilodalton antiangiogenic factor (Vstat120), can suppress angiogenesis in a CD36-dependent manner, leading to it antagonizing the neovascularization response of endothelial cells both in vitro and in vivo. [82]. Another study showed that vasculostatin is critically dependent on the presence of CD36 on endothelial cells both in vitro and in vivo, pursuing its anti-angiogenic effect [82].

CD36 is a critical receptor for regulating lipid metabolism in tumor microenvironment cells and promoting tumor growth and metastasis; therefore, CD36 may become a potential biomarker for clinical diagnosis/prognosis as well as a target for cancer therapy.

### 2.3. Thrombospondin-1

Thrombospondins (TSP-1–TSP-5) are proteins found in the extracellular matrix (ECM), with multiple effects mediated by interaction with various cell receptors, but also with cytokines, growth factors, proteases, and other stromal cell proteins [83,84]. The focus of this review is TSP-1, as a common denominator of CD36 and CD47 activation, and also due to its role in chronic inflammation, leading to angiogenesis and carcinogenesis when the homeostasis is not restored [85].

**Figure 1:**
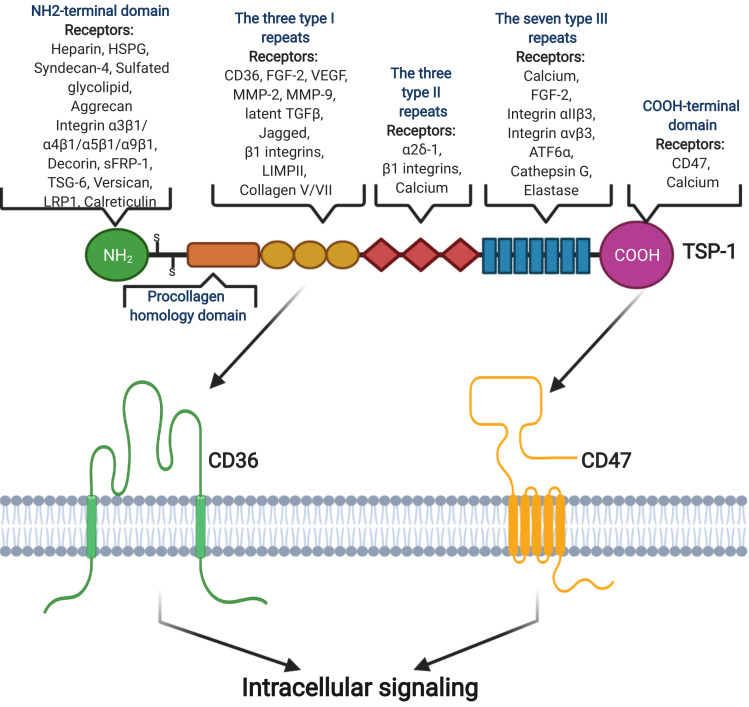
Structural domain of TSP-1 and its interactions with some receptors and ligands, although it is still unknown how TSP-1 interacts with different ligands. TSP-1 has binding sites for many inflammatory factors, such as CD47 and CD36. Image created with BioRender.com (accessed on 16 December 2020).

Recent studies identified 83 TSP-1 ligands, such as low-density lipoprotein receptor-related protein (LRP), proteoglycans and sulfatides, CD36, CD47, and CD148 [83,86] (Figure 1). The affinity of various ligands for different domains of TSP-1 results in different outcomes. For example, the NH2-terminal domain is known as a stimulator of angiogenesis [87]; the three type I repeats represents an important inhibitor of angiogenesis [88], the three type II repeats (EGF-like type II repeats) can have a major effect on the central nervous system [89], and the seven type III repeats combined with FGF-2 is active in inhibiting angiogenesis [90].

The TSP-1 role in glioblastoma is very controversial. In a recent study, Daubon T et al. showed that TSP-1 expression is higher in high-grade glioma patient samples, compared with low-grade gliomas, and TGFβ1 regulates TSP-1 expression via SMAD3-binding sites. They also observed that TSP-1 is expressed in tumor cells and vessels and tumor-derived TSP-1 is involved in GBM invasion and expansion. Anti-angiogenic treatment increased the TSP-1 expression through hypoxia-induced TGFβ1 via CD47 activation [91].

Even though TSP-1 levels are typically low in the adult brain, activated microglia and reactive astrocytes express this protein to promote neurite growth and regeneration [92]. In GBM, the number of TAMs (tumor-associated macrophages/microglia) can be very high and constitute up to 30% of the tumor mass. Though TSP-1 has been shown to reduce GBM growth and vascularity [93], the roles of TSP-1 are complex. Thus, upregulating the TSP-1 expression, especially in microglia, by blocking the EV-mediated convey of WT1 from GBM to inhibit angiogenesis might prove a good anti-GBM therapeutic strategy [94]. Conversely, Tenan M et al. demonstrate that anoxia can decrease the expression of the angiogenesis inhibitor TSP-1 and thus promote angiogenesis, and an amplified expression of TSP-1 in an in vivo model shows that even a modest reduction in TSP-1 production could be related to tumor progression [95].

The relation between p53 and TSP-1 was also investigated in glioblastomas. It has been shown that p53 does not have an effect on TSP-1 in glioblastoma [95], whereas another study determines that p53 promotes TSP-1 expression in glioblastoma [96]. The inconsistency is most likely caused by experimental differences.

Other studies compared the whole TSP-1 protein with each TSP-1 active peptide, and it has been observed that various TSP-1 active peptides may function differently. Thus, the three type I repeats (3TSR) inhibit tumor growth in glioblastoma [97], whereas the fragment 167–569, a thrombospondin-1 active peptide, which includes the procollagen homology domain and 3TSR, stimulates tumorigenicity, although it inhibits neovascularization [98]. Therefore, the procollagen homology domains may contain a cancer-promoting sequence that masks the tumor-suppressing effect of 3TSR for glioblastoma.

In glioblastoma, slight differences (*p* < 0.1) have been found in TSP-1 levels in serum from patients compared to healthy subjects, while significant differences were found when TSP-1 levels were compared in patients before and after surgery. Therefore, serum TSP-1 could be a prognostic biomarker of longer survival in patients after tumor resection [99].

Qi C et al. demonstrated that TSP-1 may be a biomarker for glioma malignancy and predict the mesenchymal subtype of GBM; they performed comprehensive bioinformatics analysis. Analyzing the correlations between THBS1 expression and immune signatures, the results reported positive correlations between THBS1 expression and Treg (a subpopulation of T cells that regulate the immune system) signatures, backing the hypothesis that THBS1 could enhance local immune tolerance in GBM [100].

Additional research regarding the functions of TSP-1 active peptides and their reasonable application is necessary. Besides mediating carcinogenesis, TSP-1 is also affected by cancer development, as reflected by its expression in plasma and the cancer tissue. Consequently, TSP-1 can be a potential biomarker for both pre-clinical and clinical applications [86].

### 2.4. CD47

CD47 represents a 50-kilodalton integral membrane protein, a member of the immunoglobulin superfamily, heavily glycosylated and expressed by most cells [101]. CD47 consists of an N-terminal extracellular IgV domain, a five-times transmembrane-spanning domain, and a short variably spliced cytoplasmic tail [101]. O-linkage of glycosaminoglycans to the IgV domain favors TSP-1 signaling through CD47 [102].

Expressed on a wide variety of normal and tumor cells, TSP-1 exhibits controversial functions in the tumor microenvironment, most of them revolving around the anti-angiogenic versus vascular apoptotic effects [85].

CD47 was reported as highly expressed in glioma cells and glioma stem cells, directly related to cell growth and differential potential. The CD47 blockade inhibited tumor growth and prolonged survival in immunocompetent mouse glioma models [103].

There are several mechanisms responsible for CD47 involvement in tumor progression. Overexpression of CD47 has been reported to have critical implications in tumor cells evasion from macrophage-mediated phagocytosis. TAMs represent the key cells responsible for an immunosuppressive tumor microenvironment by exhibiting different patterns of anti- and pro-tumorigenic phenotypes (M1/M2) [104]. The M1 phenotype is capable of releasing a cascade of proinflammatory cytokines, activating phagocytosis [105]; the impact of CD47 blockade was demonstrated to reside in its capability of shifting the TAM phenotype towards the anti-tumorigenic M1 subtype [104]. CD47-mediated immune evasion relies on its different cellular functions; however, many studies have mostly focused on its interaction with the signal-regulatory protein alpha (SIRPα), whose downstream effect of phosphorylation is the inhibition of tumor cell phagocytosis. The CD47-SIRPα axis, on the other hand, functions as a “negative immunological checkpoint,” suppressing phagocytosis by conveying the anti-phagocytic “don’t eat me” signal [106]. CD47 is known to suppress a diverse range of “pro-engulfment signals” on different target cells, such as immunoglobulin G, complement, or calreticulin (CALR), while CD47-SIRPα signaling axis is only one of the mechanisms behind its phagocytic behavior [107]. Disrupting CD47- SIRPα axis via anti-CD47 antibodies showed promising results, as different studies have revealed. Willingham et al. showed that administration of B6H12, an anti-CD47 antibody, to mice carrying human xenograft tumors, including glioblastoma, resulted in increased phagocytosis of tumor cells by macrophages that exerted a SIRPα murine form that was incapable of binding CD47. Moreover, the CD47-SIRPα axis blockade caused enhanced anti-tumor phagocytosis of TAMs, demonstrating their potential as anti-tumor effectors [108]. Gholamin S. et al. used patient-derived orthotopic xenograft models of pediatric glioblastoma and other highly malignant pediatric brain tumors to analyze the expression of CD47 on tumor cells by flow cytometry. In addition to increased CD47 expression, they also reported overexpression of CALR. Following treatment with an anti-CD47 antibody, they also reported inhibition of tumor growth, sustained by enhanced pro-phagocytic signals and probably by restimulating anti-tumor T cells, as well. Although the CD47- SIRPα axis blockade revealed beneficial effects in the struggle against brain tumors, additional pro-phagocytic stimuli are required for complete eradication of the tumor [109].

CALR was recognized as a dominant pro-phagocytic signal, overexpressed in different cancers, including glioblastoma, presenting a neglectable expression in most normal cells [110]. The pro-phagocytic signals are triggered by CALR ligation to its receptor on the surface of macrophages, LRP (low-density lipoprotein-related protein), this ligation being counterbalanced by the anti-phagocytic signals expressed via the CD47-SIRPα axis [110]. The increased expression of both CD47 and CALR on tumor cells surface raised the hypothesis that, on one hand, CALR is essential for anti-CD47 antibody-mediated phagocytosis and, on the other hand, increased CD47 expression prevents tumor cells from CALR-mediated phagocytosis, contributing to pro- and anti-phagocytic balance underlying the immune evasion mechanism [110].

Phagocytosis modulation via the CD47 blockade alone proved to have limited anti-tumor effects, with only a slight increase of phagocytosis in human GBM cells. By combining anti-CD47 antibody with temozolomide chemotherapy, an increase in CALR translocation to the cell surface was observed, associated with an enhanced pro-phagocytic effect on tumoral cells [111]. The therapeutic use of CD47 blockade combined with either temozolomide or irradiation enhanced its effects by increasing macrophage-mediated phagocytosis of GBM cells, resulting in an increased survival rate of GBM-implanted mice and a significant inhibition of tumor growth. It was concluded that various mechanisms could be involved for the enhanced response, and the anti-CD47 antibody used in this study was responsible for blocking the CD47- SIRPα axis [112].

It was also revealed that CD47 ligation recruits and affects SIRPα localization, and its subsequent phosphorylation induces its repositioning at the phagocytic synapse site, which in turn inhibits integrin signaling in macrophages, limiting and suppressing phagocytosis as a downstream effect. Similar to CD47-blocking antibody, suppressing integrin activation has consequently limited the macrophage dissemination on the surface of tumor cells, allowing tumor cells to evade phagocytosis [107].

Immune evasion of tumor cells by phagocytosis disruption via CD47-mediated blockade turned out to have critical roles in cancer immunotherapy, CD47 representing an “immune checkpoint molecule” whose therapeutic modulation could unravel promising strategies for further clinical applications, and its expression level could be considered as a relevant prognostic marker in gliomas [106,108,113].

## 3. CD36 and Thrombospondin-1 in Glioblastoma

Cooperation between TSP-1 and CD36 was studied in relation to their ability to induce endothelial cell apoptosis and thus to inhibit neoangiogenesis within tumors [85,114,115]. TSP-1 binding to CD36 activates downstream Src kinases such as Fyn. CD36–TSP-1- Fyn axis promotes increased phosphorylation level of Fyn followed by activation of p38/caspase-3 and elevated expression of endogenous proapoptotic receptors (FAS, TNF receptor, and TRAIL (TNF-related apoptosis-inducing ligand) receptors death receptor 4 and 5). A soluble trimeric form of TRAIL (sTRAIL) has been shown to have an important selective anti-GBM role [116]. The particular heterogeneity of GBM has been a setback for potential treatment targets due to TRAIL resistance mechanisms. Recently, studies showed a promising counter-fight: mesenchymal stem cells -3TSR/s-TRAIL inhibiting effects over GBM progression by modulating both tumor cells and endothelial cells [97].

Another pathway by which TSP-1 modulates microvessel formation is by blocking vascular endothelial growth factor (VEGF) signaling, therefore stopping tumor progression [117]. Moreover, VEGFR2 signaling was shown to be inhibited in a CD 36-dependent manner through Src homology 2 domain-containing protein tyrosine phosphatase 1 (SHP-1). This phosphatase dephosphorylates the VEGFR2 signaling complex, reducing VEGF signal transduction [118].

The discovery of histidine-rich glycoprotein (HRG), a protein released in plasma after platelet activation which acts as a decoy receptor for TSP-1, could offer a promising insight into the angiogenic switch and a potential treatment target for GBMs. HRG interference with TSP-1/CD36 binding is explained by preferentially binding to TSP-1 to the detriment of CD36 (through a receptor domain mimic) and thus promoting a proangiogenic effect [81,119]. Blocking HRG promotes endothelial cells CD36 receptor binding of angiostatic protein, vasculostatin, therefore promoting an anti-angiogenic effect by initiating the apoptotic pathways [119,120].

## 4. CD47 and Thrombospondin-1 in Glioblastoma

CD47-TSP-1 axis is of interest for GBM for its anti-angiogenetic effect, which may be exerted through several different pathways. One of them is the antagonizing of the NO signal transduction pathway [121], a known pro-angiogenic factor [122].

The other is the VEGF signaling pathway, as CD47 is known to mediate the inhibition of the TSP-1/VEGF signaling pathway [102,123] by directly binding to VEGF, competing for VEGF binding to heparan sulfate proteoglycans on microvascular endothelial cells and by generating inhibitory signaling through the TSP-1 receptors, such as CD36 and CD47 [118], or even by regulating the expression of VEGF and VEGFR2 [124]. Of these mechanisms, only the inhibition of CD47 signaling has been validated at physiological subnanomolar concentrations of TSP-1 [125,126,127].

Furthermore, the TSP-1/CD47 signaling pathway has an anti-inflammatory effect, by inhibiting the activation of NF-*κ*B/AP-1 [128].

## 5. Signaling Pathways Activated by CD36 and CD47 Binding to TSP-1 in Glioblastoma

**Figure 2:**
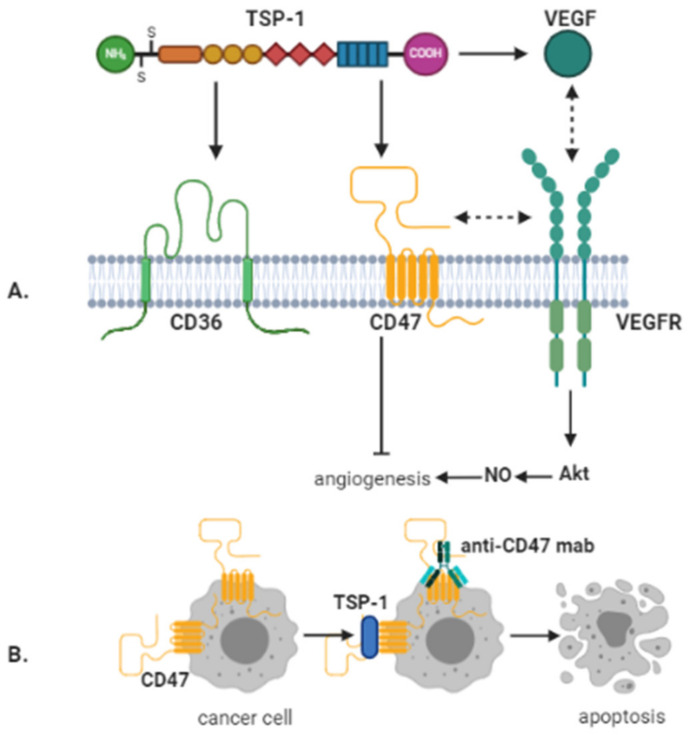
Anti-angiogenic and direct anti-cancer effects mediated by CD47. (**A**) TSP-1 inhibits angiogenesis via binding to CD36 and CD47. However, TSP-1 mediated inhibition of angiogenesis by binding to CD36 is also regulated via CD47. In addition, CD47 directly interacts with vascular endothelial growth factor receptor-2 (VEGFR-2) on endothelial cells. By binding to CD47, this interaction is abrogated by TSP-1, whereby angiogenesis is inhibited. Further, TSP-1 can directly bind to VEGF, thereby preventing its interaction with VEGFR-2. (**B**) Crosslinking of CD47 by antibodies or TSP-1 can lead to caspase-independent cancer cell death. Image created with BioRender.com (accessed on 14 January 2021).

As already mentioned, TSP-1 is an endogenous inhibitor of angiogenesis, as a negative regulator of NO-mediated signaling in endothelial cells, vSMC, and platelets [129,130]. TSP-1 inhibition of NO/cGMP-related signaling pathways and consequent anti-angiogenic activities are facilitated by its interaction with two cell surface receptors: CD47 and CD36 [126,131]. TSP-1 anti-angiogenic action was at the beginning related to the binding to CD36 expressed on endothelial cells [132]. TSP-1 is now known also to interact with CD47 through its COOH terminal domain [133] for specific roles in tumorigenesis, particularly cell motility [85,117,134]. Even more so, CD47 is essential for the anti-angiogenic effect of TSP-1, as CD47-/- cells failed to enter apoptosis following TSP-1 stimulation (Figure 2B). This was not observed for CD36-/- cells, while the coupling of CD36 failed to inhibit the proliferation of CD47-/- NO stimulated cells. Moreover, the anti-angiogenic effects induced by TSP-1 were blocked by a TSP-1 peptide recognizing CD47, thus conducive to the inhibition of vascular outgrowth. Moreover, the ligation of TSP-1 on CD47 was efficient to block the responses stimulated by NO on vascular cells [135]. Thus, TSP-1 mediated inhibition of angiogenesis is regulated via interactions with both CD36 and CD47, whereby CD47 likely acts downstream of CD36 in endothelial signaling. Therefore, CD47 is the dominant anti-angiogenic receptor for TSP-1 mediated inhibition of angiogenesis (Figure 2A).

VEGF-VEGFRs axis is another junction point for TSP-1/CD36/CD47 signaling. Overall, the effect of TSP-1/CD36/CD47 joint activity on VEGF signaling is inhibitory on various levels: downstream inhibition of Akt [136], dephosphorylation, and subsequent inactivation of VEGFR2 [118] or direct binding of TSP-1 to VEGF, preventing the activation of VEGFR [137].

At micromolar concentrations, TSP-1 can inhibit VEGF expression by VEGFR2 phosphorylation after CD36 ligation. At nanomolar concentrations, TSP-1 can also inhibit VEGF signaling directly or by competing for binding with VEGF. In contrast, a high endogenous picomolar concentration of TSP-1 is stimulating VEGF, suggesting a direct interaction between these two receptors: CD36 and VEGFR2 (Table 1).

Inflammation and neoplasia lead to elevated TSP-1 levels [85,135]. Therefore, evidence suggests that other receptors in addition to CD47 are activated, e.g., 100-fold greater concentrations of TSP-1 triggers CD-36 signaling pathway [125,126,127].

Lipid rafts and tetraspanin-enriched microdomains (αv, α5, β1, and β2 integrins) are mentioned as part of the structure of CD36 receptor [140,141,142]. TSP-1 binds the integrins expressed on apoptotic neutrophils and CD36 expressed on macrophages [143]. Under the stimulation of TSP-1, VEGFR2 dissociates from CD47 and binds to CD36, thus promoting VEGFR2 dephosphorylation in the presence of integrin/SHP1 complex [125]. It has been confirmed by Primo et al., 2005 that this effect is blocked by mutating CD36 in order to hinder β1 integrins association [144].

## 6. Therapeutic Targeting of TSP-1/CD36/CD47

Knowledge regarding TSP-1 interaction with CD47 and CD36 has been translated towards clinical research. There are several axes that were effectively targeted as therapeutic strategies, based on blocking CD36 and CD47 expression or activation on different levels [84,145].

Blockage of activation can be achieved using several strategies. One direction is the usage of monoclonal antibodies (mAbs), which are developed in different stages of clinical trials [146,147]. The immune checkpoint role of CD47 is being exploited in early phase clinical trials by developing dual antibodies, which will also bind to PD-L1 to increase the anti-tumor efficiency of the immune response [148]. Another strategy is the development of derived peptides from TSP-1 (4N1/4N1K; 7N3, PKHB1-containing a critical VVM motif suggested to be critical for CD47 binding) that target CD47 or derived peptides from CD47 (TAX2) that target TSP-1.

It is debatable whether 4N1/4N1K may be considered a CD47 agonist due to some studies which mentioned that 4N1K mediated biological effects independent of CD47 [149,150]. Multiple administrations of 4N1K peptide induce 30% inhibition of tumor growth in treated mice compared with control mice, an effect which was associated with a decrease in the Ki67-positive cells number [151]. Afterward, Leclair P et al. in 2014 mentioned the predilection of 4N1K to bind non-specifically to proteins, revealing that this behavior had a significant role in the 4N1K-mediated cellular effects [152]. In conclusion, caution should be observed when interpreting 4N1K- or PKHB1-induced cell phenomena.

TAX2 is a cyclic 12 amino-acids peptide generated from CD47, which targets TSP-1 and selectively prevents TSP-1:CD47 interaction. Administrations of TAX2 induce significant tumor necrosis in syngeneic melanoma models and also reduce the growth rate and vascularization of human pancreatic carcinoma xenografts in nude mice [153]. Daubon et al. demonstrated that in glioblastoma tumors, TAX2 as an individual treatment reduces the vascular density of orthotopic tumors and presented inhibitory activity in vitro under hypoxia in P3 cells and impaired single-cell invasion in vivo. Combined with bevacizumab, TAX2 inhibits contralateral tumor invasion in the same tumor model [91]. Jeanne A et al. in 2021 showed that in vivo TAX2 treatment reduces ovarian tumor development and metastasis while activating anti-cancer adaptive immunity [154].

TSP-1 recombinant fragment: 3TSR (TSP-1 type I repeats) or 3TSR/TRAIL (TSP-1 type I repeats/TRAIL) fusion protein represent another therapeutic strategy. Depending on the TSP-1 sequence, the mimetic peptide may inhibit CD47 or CD36, but the clinical endpoints are usually similar—survival period and secondary effects. There is also a non-peptide small molecule (sm) mimicking the FGF-2 binding site located in the type 3 repeats of TSP-1 [155] that exhibits in vitro and ex vivo antiangiogenic properties [156]. Numerous computational studies have been conducted to improve sm: FGF-2 binding dynamics [157,158], and newly designed derivatives will presumably be evaluated in vivo in further experiments. Given the combined action of CD36 and CD47, a compound interfering with both receptors (VT1021, a cyclic pentapeptide) was tested for safety in solid tumors, including glioblastoma [159]. A third direction being developed in clinical trials is the prevention of CD47/SIRPα activation. The usage of a soluble recombinant fusion protein encoding the N-terminal CD47 binding domain of human SIRPα was proven effective as anti-tumor therapy for selected hematopoietic malignancies and several solid tumors, including glioblastoma (reviewed in [160]). Velcro-CD47 (N3612) consists of a high-affinity variant of the human CD47 extracellular domain extended at the N-terminus with a short three-amino-acid peptide that increases binding affinity to SIRPα [161]. Velcro-CD47 already proved its ability to enhance macrophage phagocytosis of tumor cells in vitro as well as to target the monocyte subpopulation specifically, and its putative anticancer efficacy will be assessed in further pre-clinical models.

Downregulation of CD47 protein expression can be achieved by antisense nucleotides, preventing CD47 mRNA translation to protein, but this direction of research is also in preclinical testing.

## 7. Conclusions

GBM, a highly aggressive and heterogeneous tumor, is characterized, like other aggressive tumors, by altered lipid metabolism. This rewiring, aiming to increase the energetic output, is achieved, among other mechanisms, with the help of FA transport proteins, such as CD36. Known also as a scavenger receptor, CD36 can bind to various ECM molecules, activating intracellular signaling relevant for inflammation, macrophage-related immune responses, and cell survival. One of its ECM binding partners, TSP-1 is also recognized by CD47, a cell receptor member of the immunoglobulin superfamily. Numerous data offered significant evidence that both CD36 and CD47 converge by binding to TSP-1, towards an anti-angiogenic effect, which points to this triad as effective adjuvant therapy for aggressive solid tumors, such as GBM. So far, although both receptors are targeted separately in clinical practice by different methods, like fully-humanized monoclonal antibodies or TSP-1 mimicking peptides, no joint targeting has been reported until recently. A novel peptide compound, VT1021, with dual effect has been recently proven safe in clinical trials and will be advancing to efficacy testing against solid tumors, including GBM. Hopefully, this effort will open the avenue for a new class of effective therapies against GBM.

## Figures and Tables

**Table 1:** TSP-1 concentration effects.

TSP-1 Concentration	Ligand	Signaling Pathway/Proteins	Effect
**10–100 pM**	**CD47**	NO/cGMP/cGK	Increased endothelial cell adhesion [125,126,131,135]
		eNOS/NO/sGC	Anti-angiogenic effect/inhibits NO production [127,131]
**100–150 pM**	**CD47**	VEFGR2 direct binding	Anti-angiogenic effect [125,138]
**1–10 nM**	**CD36**	NO/cGMP/cGK Src kinasesFyn/p38 phosphorylation	Inhibits NO production Anti-angiogenic effect [126] Endothelial cell apoptosis [139]

## Data Availability

Not applicable.
